# Solidarity and strife after the Atlanta spa shootings: A mixed methods study characterizing Twitter discussions by qualitative analysis and machine learning

**DOI:** 10.3389/fpubh.2023.952069

**Published:** 2023-02-07

**Authors:** Shaniece Criss, Thu T. Nguyen, Eli K. Michaels, Gilbert C. Gee, Mathew V. Kiang, Quynh C. Nguyen, Samantha Norton, Eli Titherington, Leah Nguyen, Isha Yardi, Melanie Kim, Nhung Thai, Ariel Shepherd, Chris J. Kennedy

**Affiliations:** ^1^Department of Health Sciences, Furman University, Greenville, SC, United States; ^2^Department of Epidemiology and Biostatistics, University of Maryland School of Public Health, College Park, MD, United States; ^3^Division of Epidemiology, School of Public Health, University of California, Berkeley, Berkeley, CA, United States; ^4^Department of Community Health Sciences, University of California, Los Angeles, Los Angeles, CA, United States; ^5^Department of Epidemiology and Population Health, Stanford University School of Medicine, Stanford, CA, United States; ^6^Department of Public Health Science, University of Maryland, College Park, MD, United States; ^7^Department of Anthropology, Brown University, Providence, RI, United States; ^8^Department of Nutritional Sciences and Toxicology, University of California, Berkeley, Berkeley, CA, United States; ^9^College of Arts and Sciences, University of South Carolina, Columbia, SC, United States; ^10^Department of Psychiatry, Harvard Medical School, Boston, MA, United States

**Keywords:** anti-Asian racism, Twitter, machine learning, qualitative content analysis, solidarity

## Abstract

**Background:**

On March 16, 2021, a white man shot and killed eight victims, six of whom were Asian women at Atlanta-area spa and massage parlors. The aims of the study were to: (1) qualitatively summarize themes of tweets related to race, ethnicity, and racism immediately following the Atlanta spa shootings, and (2) examine temporal trends in expressions hate speech and solidarity before and after the Atlanta spa shootings using a new methodology for hate speech analysis.

**Methods:**

A random 1% sample of publicly available tweets was collected from January to April 2021. The analytic sample included 708,933 tweets using race-related keywords. This sample was analyzed for hate speech using a newly developed method for combining faceted item response theory with deep learning to measure a continuum of hate speech, from solidarity race-related speech to use of violent, racist language. A qualitative content analysis was conducted on random samples of 1,000 tweets referencing Asians before the Atlanta spa shootings from January to March 15, 2021 and 2,000 tweets referencing Asians after the shooting from March 17 to 28 to capture the immediate reactions and discussions following the shootings.

**Results:**

Qualitative themes that emerged included solidarity (4% before the shootings vs. 17% after), condemnation of the shootings (9% after), racism (10% before vs. 18% after), role of racist language during the pandemic (2 vs. 6%), intersectional vulnerabilities (4 vs. 6%), relationship between Asian and Black struggles against racism (5 vs. 7%), and discussions not related (74 vs. 37%). The quantitative hate speech model showed a decrease in the proportion of tweets referencing Asians that expressed racism (from 1.4% 7 days prior to the event from to 1.0% in the 3 days after). The percent of tweets referencing Asians that expressed solidarity speech increased by 20% (from 22.7 to 27.2% during the same time period) (*p* < 0.001) and returned to its earlier rate within about 2 weeks.

**Discussion:**

Our analysis highlights some complexities of discrimination and the importance of nuanced evaluation of online speech. Findings suggest the importance of tracking hate and solidarity speech. By understanding the conversations emerging from social media, we may learn about possible ways to produce solidarity promoting messages and dampen hate messages.

## Introduction

On March 16, 2021, a white man shot and killed eight victims, six of whom were Asian women (1). The victims were Daoyou Feng, Hyun Jung Grant, Suncha Kim, Paul Andre Michels, Soon Chung Park, Xiaojie Tan, Delaina Ashley Yaun, and Yong Yue. The murders took place in a massage parlor and two different spa salons, places that the shooter described as “temptations he wanted to eliminate” as a method to address his sex addiction (2). This event occurred during a period of rising concern about anti-Asian racism, sparking outrage across the country and evoking fear in the Asian American community. In response, communities held vigils and organized rallies to remember the victims and called people to take action.

Set against the increased hate crimes committed against Asian Americans and Asians living in America during the COVID-19 pandemic (3, 4), this tragic massacre brought more attention to the racism experienced by Asian Americans and sexualization of Asian American women in particular (5). Asian American women working in spa salons or massage parlors are often assumed to be sex workers, which incites fetishization, objectification and racial sexualization (6–8). The racial sexualization of Asian women dates back to the 1800s (7) and has persisted into present day, with Asian and Asian American women often fetishized as being “exotic” creatures that are small, cute, and submissive (5, 8–10).

The COVID-19 pandemic ushered in a rise in anti-Asian sentiment and hate crimes (11, 12). Reports of anti-Asian physical assaults rose from 10.2 to 16.7% between 2020 and 2021, and online hate incidents increased from 5.6 to 10.2% (3). The spike in hate crimes against Asian Americans during the pandemic has been associated with an increase in the usage of terms for coronavirus such as “Chinese virus” and “Wuhan virus” (13). The spread of the usage of “Chinese virus” was catalyzed by highly influential political figures on social media (14). Attaching ethnicity and race to SARS-CoV-2, the causative agent of COVID-19, increased stigma against Asian Americans and Asians around the world (13, 15, 16).

In tandem, the police murders of George Floyd, Ahmaud Arbery, Breonna Taylor, and other unarmed Black Americans in 2020 ignited a nationwide discussion about race and racism (17). With the confluence of racial reckoning in the US and the COVID-19 pandemic, the timing of the Atlanta spa shootings renewed conversations of the role of racism in this incident and beyond. An understanding of the quality and quantity of public discourse surrounding race and racism in the United States following this tragic event can provide important insights to initiate interventions to protect marginalized groups and galvanize change during moments of heightened awareness of social injustice (18).

Twitter provides insight into the public's reaction to racialized events (13, 17). A 2016 Pew Research Center study found, over a 15-month period, 60% of tweets discussing race were connected to a current race-related event (17). In past studies, Twitter data have been used to analyze perspectives about race and the COVID-19 vaccine (19), the use of stigmatizing COVID-19 terms and Anti-Asian sentiment (11, 13), the aftermath of the shooting of Michael Brown (20), and the killings of Ahmaud Arbery, Breonna Taylor, and George Floyd (18). Following the Atlanta spa shootings, there was an outpouring of public support expressed on Twitter, with trending hashtags such as #StopAsianHate. Examining discourse related to the Atlanta spa shootings provides a unique opportunity to examine trends in themes in speech toward the Asian American community in the aftermath of this tragic event. Therefore, the present study aimed to: (1) describe qualitative themes of tweets related to race, ethnicity, and racism immediately following the Atlanta spa shootings, and (2) examine temporal trends in expressions of a new measure of hate speech and solidarity before and after the Atlanta spa shootings using a new methodology for hate speech analysis.

## Methods

### Overview

We used a mixed-methods approach that integrated qualitative content analyses with state-of-the art machine learning analysis of race-related publicly available tweets. Qualitative content analyses provided in-depth understanding of the themes, and machine learning assessed national trends in the data as well as quantified hate speech and solidarity speech. A random 1% sample of publicly available tweets was collected from January 2021 to April 2021 using Twitter's Streaming Application Programming Interface (API). Details of the data collection process including the full keyword list are available (21). We restricted our analyses to English language tweets from the US that used one or more of 518 race-related keywords compiled from racial and ethnic categories used by prior studies examining race-related online conversations (22) and an online database of racial slurs. Tweets were classified into four main racial/ethnic categories: Asian, Black, Latinx, and White according to the keywords used. For this paper, we focus on tweets referencing Black and Asian people. The 202 keyword list for Asian and Black related tweets are presented in Supplementary Table 1. The analytic sample included 708,933 tweets.

### Content analysis

Through content analysis we sought to understand the national discussion before and after the Atlanta spa shootings on March 16, 2021. Our qualitative content analysis provides information about observed trends, topics, and themes to understand this time period. Our team has been continually collecting Twitter data since 2015 to examine changes in racial attitudes over time. As a result, we had access to tweets before the incident and used them to establish a baseline. Our study analysis had a random sample of 1,000 tweets referencing Asians preceding the Atlanta spa shootings (January 1 to March 15, 2021) and 2,000 tweets referencing Asians after the shootings (March 17 to March 28, 2021) to examine potential temporal changes in discussion topics and sentiment.

The team developed the codebook based on a literature review and preliminary analysis of the first 200 tweets from the sample to solidify the codes and their definitions. The codes acted as broad categories. The final codes were: (1) solidarity, (2) condemning shootings, (3) racism, (4) role of racist language during the pandemic, (5) intersectional vulnerabilities, (6) relationship between Asian and Black struggles against racism, and (7) common discussions (tweets not relevant to the spa shootings incident). The next 600 tweets were triple-coded by three study team members independently, and the remaining 1,200 tweets were double-coded by two study team members independently. Six members of the study team discussed all discrepancies in the coding and came to a consensus on the final code for each tweet. The counts were reported for each code category. Once all the tweets were coded, the qualitative analysts met to discuss and finalize specific themes within each code. Utilizing thematic analysis, the team analyzed tweets within each code to identify themes (23). There were multiple themes highlighted within each code category that provided nuanced meaning about online speech pertaining to race and ethnicity. With this extensive consensus-building process, we sought to maintain data trustworthiness through utilizing multiple data analysts from various geographic regions of the US and who identify as Asian, Black, and White.

### Hate speech measure, a new machine-learning methodology

We also employed a supervised machine learning to quantitatively analyze the sentiment of 708,933 tweets. Machine learning uses algorithms and models to present patterns from the data, and the hate speech methodology is a type of machine learning. Our recently developed method combines faceted item response theory (IRT) with deep learning to measure hate speech on a continuous, interval spectrum. Details on the development of the hate speech measure are documented (24). The construct of hate speech was operationalized as a composite of nine simpler phenomena (components) that could be labeled as ordinal survey items by human reviewers: sentiment, respect, insult, humiliation, status, dehumanization, violence, genocide, and attack-or-defense. A 10th component for hate speech itself (yes/no/unclear) was included for benchmarking purposes. Solidarity speech was defined as hate speech scores <-3 and racist speech was defined as hate speech scores >0.5. Each tweet was further scored on the components of hate speech.

Supervised machine learning models, such as our hate speech model, require training data to understand how a human reviewer would rate the tweet. The training dataset—consisting of 50,000 social media comments sourced from YouTube, Twitter, and Reddit—was labeled by 10,000 United States-based Amazon Mechanical Turk workers on those components of hate speech (the dataset is available at https://huggingface.co/datasets/ucberkeley-dlab/measuring-hate-speech) (25). Amazon Mechanical Turk is a crowdsourcing marketplace where tasks can be performed virtually. Table 1 shows the questions from the annotation guide. The crowdsourced labels were combined *via* a non-linear IRT scaling transformation into a continuous outcome measure, yielding an interval-valued spectrum ranging from violent hate speech on one extreme (+5.0) to supportive identity speech on the other (−8.0). During the scaling process, the IRT model simultaneously estimates and eliminates the interpretation bias of the human labelers. The response quality of each individual labeler was also estimated using the IRT model, allowing responses from low-quality labelers to be removed (30% of labelers). The IRT scaling procedure was then integrated with a multitask deep learning model based on the RoBERTa-Large language representation architecture for automated prediction on new data, which estimates both the continuous hate score and each of the constituent components (26). The resulting model achieved a cross-validated correlation of 84% and mean absolute error of 0.85 at predicting the continuous hate speech score. This model is far more accurate than the 66% correlation and 1.7 mean absolute error of Google Jigsaw's Perspective API models, which is possibly the most widely used hate speech detector (27). This novel hate speech measurement system allowed us to estimate the precise location of each tweet on the hate speech spectrum, where higher scores were more indicative of violent, racist language, and lower scores were indicative of benevolent race-related speech.

**Table 1:** Components of hate speech with their associated annotation prompts and labels.

**No**.	**Component**	**Annotation prompt (abridged)**	**Labels**
1	Sentiment	How would you describe the sentiment of this comment?	Strongly negative—strongly positive
2	Respect	Is this comment respectful toward the group(s) you previously identified?	Strongly disrespectful—strongly respectful
3	Insult	Comment is insulting toward the group(s) you previously identified.	Strongly disagree—strongly agree
4	Humiliate	Comment is humiliating toward the group(s) you previously identified.	Strongly disagree—strongly agree
5	Status	Comment states that the group(s) you previously identified is	Strongly inferior—strongly superior
6	Dehumanization	Dehumanizes the group(s) you previously identified (e.g., by comparing them to an animal)	Strongly disagree—strongly agree
7	Violence	Calls for using violence against the group(s) you previously identified	Strongly disagree—strongly agree
8	Genocide	Calls for the deliberate killing of a large group of people from the group(s) you previously identified.	Strongly disagree—strongly agree
9	Attack-defend	Attacking or defending the group(s) you previously identified	Strongly defending—strongly attacking
10	Hate speech benchmark	Contain hate speech, defined as bias-motivated, hostile, and malicious language targeted at a person/group because of their actual or perceived innate characteristics, especially when the group is unnecessarily labeled	Yes, no, unsure

## Results

### Qualitative results

Table 2 provides the categories, themes, and illustrative tweets arising from the content analysis. The category and themes are described below. Qualitative themes that emerged included solidarity (4% before the shootings vs. 17% after), condemnation of the shootings (9% after), racism (10% before vs. 18% after), role of racist language during the pandemic (2 vs. 6%), intersectional vulnerabilities (4 vs. 6%), relationship between Asian and Black struggles against racism (5 vs. 7%), and discussions not related (74 vs. 37%).

**Table 2:** Content analysis themes of tweets related to race/ethnicity before and after the Atlanta spa Shootings (with illustrative examples).

**Before the shootings**	**After the shootings**
**Example tweets from time period 1—January 1 to March 15, 2021:** ***n*** = **1,000 tweets**	**Example tweets from time period 2—March 17 to March 28, 2021:** ***n*** = **2,000 tweets**
**Themes** **Solidarity**	
4% of the sample (*n* = 46)	17% of the sample (*n* = 339)
–	**Sympathy and support** • My beautiful Asian Americans, I'm so sorry. I stand with you.
–	**Community collectiveness** • STOP ASIAN HATE rally early this morning here in San Francisco Downtown! All Asians unite, young and old—so amazing to see Asians united.
**Affirmations** • The Chinese kinda rock	–
**Celebrations** • I heard that in support of the Asian community, folks was celebrating Chinese New Year last night downtown with some fireworks.	–
**Call for action** • Racist-fueled attacks on Asian American communities must end NOW! Spread positivity or I pray you run into the right one.	**Call for action** • The continued violence directed against the Asian American Pac Island community is vile and disgusting. We cannot allow ignorance and hate to continue to spread. Bigotry and xenophobia must be called out.
–	**Interpersonal support** • I'm more than happy to accompany any of my Asian friends if they don't feel comfortable or don't feel safe going out by themselves to the store, wherever (this included rides to avoid public transportation my heart hurts so badly seeing innocent people being attacked like this)
**Condemning shootings** 0% of the sample	9% (*n* = 171)
•Not applicable	**Hate crime** • The murders of 6 Asian women is seen by many of us as a hate crime. I don't get that law enforcement doesn't see it the same way. **Grappling with personal emotions** • Terrifying. We're really upset over here. Be extra gracious with Asian people right now, because we're not okay. **Disgust in response toward the shootings (police, media, and government)** • The Asian women, and a white cop defends this murderer by saying he had a bad day? Excuse me?? #AsianLivesMatter #AsiansAreHuman #StopAsianHate #StopAsianHateCrimes
**Racism** 10% (*n* = 101)	18% (*n* = 366)
**Anti-Asian racism and attacks**	**Anti-Asian racism and attacks**
• Racist man pepper sprays Asian gas station owner after telling him to “go to China”	• A man ran full speed onto my train today because some dude was angrily yelling at the station. I couldn't even make out what he was yelling about, but the fact that the first man, who was Asian, felt so scared that this guy could attack him
**Racist insults**•Must be where the Chinese handlers are staying. Like roaches, they come out in the dark.	–
–	**White supremacy** • @[NAMES] You don't have to be white to be a white supremacist. A lot of the groups subjugated by white colonizers have people in their societies that are white supremacist identifiers or white supremacist sympathizers. This is applies to Black, Brown, and Asian people.
–	**Downplaying racism against Asian people** • Being the “model minority” there is an invisible pressure that when an Asian person is murdered, we are not allowed to mourn and protest the injustice of it. We are told being immigrants that outrage and asking for anything more than what we have isn't our culture.
**Historic racism** • Asian Americans have always dealt with the “forever foreigner” problem. I'm tired as well, but the next generation needs to change this for good	**Historic racism** • These hate crimes have not started because of increased media attention. This sh*t has been going on for years. Due to anti-Asian stereotypes there are a lot of Americans, some I know personally that are scared of eating out of any Asian restaurants because of the long-running [stereotypes]
**Language** 2% (*n* = 18)	6% (*n* = 112)
**Role of racist language and its consequences during the pandemic** • My son's gf is half Chinese and when COVID first hit, she got harassed at the grocery store 1 day with her mom and little sisters. Some as*hole threw a bottle of cleaner at them and ran away screaming “China virus they brought the China virus!” Just god awful.	**Role of racist language and its consequences during the pandemic** • This isn't fair!!This was obviously a hate crime targeting Asians & Pacific Islanders because they feel emboldened from racist, xenophobic rhetoric they absorb from right-wing media to promotes fear-mongering, racism & division to the community. #StopAsianHate #StopAAPIHate
–	**Defending the rhetoric** • I'm sorry! some people don't do logic—like that there's a difference between China, the place, the Chinese government and Chinese people in or out of China (let alone throwing in other Asians) but the virus IS from China
**Intersectional vulnerabilities** 4% (*n* = 39)	6% (*n* = 126)
**Intersectional racial violence** • I'm so sick of this sh*t. People who's attacking the elderly Asians are soft as hell. Weak! Just plain disgusting POS that would do this.	**Intersectional racial violence** • It's not just violence against Asian Americans. It's violence against the weakest Asian Americans. It's the elderly. It's women. It's the poor and marginalized. It's infuriating.
–	**Fear from intersectional identity** • I now feel more and more scared to be alone outside as an Asian woman. This shouldn't be a feeling I feel or any woman of any color should feel.
**Sexualization** • Hi guys! Good mood! Have fun f*cking! #asian #asianwomen #fetishmodel #asianbabe	–
	**Sexualization as racism**
–	• idk who needs to hear this: fetishizing Asian people *is* racism.
	**Sex addiction narrative**
–	• Premeditated murder. You have a sex addiction so you kill Asian women. That doesn't hold water
**Relationship between Asian and Black fight against racism** 5% (*n* = 53)	7% (*n* = 141)
**Friction** • Using the rise in Asian hate crimes as an excuse to be anti-black is still racism bud	**Friction** • Just saying this… where are all the Asians screaming BLM • Not noticed it's Blacks doing most of the attacking on Asian People? Screw BLM!! • This stop Asian hate propaganda is a counter movement to overshadow Black American issues America wants to bury and silence. If Black people hate Asians so much we wouldn't be their largest consumer base.
–	**Calls for solidarity** • We can support Black Lives Matter and support our Asian brothers and sisters as they deal with anti-Asian violence. Be against White supremacy, for equality. Don't stand by. Stand up. Read my take at @[NAMES]
**Multiracial observations** • I would love to see a multiracial family show that's Black and Indian or Black and Hispanic or Black & Asian because TBH that's more prevalent than the Black &White family.	–

Common discussions refer to tweets not relevant to the topic. Before the shootings, 74% of the sample (n = 743) were in this category. After the shootings, 37% of the sample (n = 745 tweets) were in this category. Most tweets in this category referred to appreciating Asian food, providing information and opinions about politics, and perspectives of popular culture related to media and entertainment.

#### Solidarity

Before the shootings: This category represented 4% of the sample and had three themes. *Affirmations* described positive compliments toward the Asian community. *Celebrations* highlighted excitement about events and people. *Call for action* tweets focused on imploring people to take a stand against attacks on Asian Americans.

After the shootings: This category represented 17% of the sample and had four themes. *Sympathy & support* described tweets of expressing sadness and “standing” with Asian Americans to offer reassurance. *Community collectiveness* were tweets that focused on solidarity through rallies and community-based resources. *Call for action* tweets focused on “calling out” hate and imploring people to take action to stop the violence. *Interpersonal support* was captured in tweets that gave specific ways to support Asian people in their personal network through supporting businesses, friends, and associates. News reports about the shootings were also captured in this section.

#### Condemning shootings

Before the shootings: The theme of condemning the shootings was not applicable to this time period.

After the shootings: This category represented 9% of the sample and had three themes. The *hate crime* theme focused on the perceived targeted attacks on Asian women and the need for the crime to be labeled as a hate crime based on the event and not the shooter's expressed motives. The *grappling with personal emotions* theme captured the negative emotions (e.g., fear, sadness, anger) of Asian people and fear for Asian loved ones in the aftermath of the shootings. After the event, tweets reported *disgust in response toward the shootings*. Specifically, tweets reported anger toward the police officer who reported that the shooter was “having a bad day” at a press conference, distrust in media coverage, and disappointment in lack of response from some elected officials.

#### Racism

Before the shootings: This category represented 10% of the sample and had three themes. These tweets highlighted recent examples of *anti-Asian racism and attacks*. *Racist insults* described users who tweeted disparaging and offensive remarks about Asian people. In this period, *historic racism* captured the trauma and resulting fatigue of racism through the generations.

After the shootings: This category represented 18% of the sample and had four themes. In connection with the shooting violence, tweets highlighted other recent examples of *anti-Asian racism and attacks* through statistics and stories of Asian people being attacked. Tweets described the role of *white supremacy* as the “root cause” of racism and impacts other minoritized groups. *Downplaying racism against Asian people* described tweets that denied or belittled racism in this group and could potentially stifle action among Asian people. Tweets also described *historic racism* by reporting generations of discrimination against Asian people through stereotypes and government mistreatment.

#### Role of racist language during the pandemic

This theme represented 2% of the sample before and 6% of the sample after the shootings. These tweets discussed *role of racist language and its consequences during the pandemic*. Several tweets discussed the stigmatizing language related to the COVID-19 pandemic in relation to people perceived as Chinese. Consequences described in the tweets included harassment and discrimination.

#### Intersectional vulnerabilities

Before the shootings: This category represented 4% of the sample and had two themes. Tweets about *intersectional racial violence* described the appalling attacks on elderly Asians, Asian women, and elderly Asian women. *Sexualization* described tweets that objectified Asian people.

After the shootings: This category represented 6% of the sample and had four themes including discussions of interracial violence observed before the shootings. After the shootings, Twitter users expressed *fear from intersectional identity* making them or someone they knew a target of violence. *Sexualization as racism* described tweets that identified the fetishizing of Asian people as a form of racism. Lastly, the shooter stated that he committed the shootings because he had a sex addiction, and the *sex addition narrative* contested that explanation as a rationale for killing people.

#### Relationship between Asian and Black fight against racism

Before the shootings: This category represented 5% of the sample and had two themes. *Friction* described the conflict between Asian and Black people. *Multiracial observations* represented users' general observations about race through informing or providing their opinion, such as lack of representation on television.

After the shootings: This category represented 7% of the sample and had two themes. *Friction* described tweets that noted the antagonism between Asian and Black people, specifically pointing out the conflict and lack of support between these groups. In contrast, other tweets highlighted *calls for solidarity* between Asian and Black people. Both themes in this category highlighted the role of white supremacy in creating tension among races.

#### Common discussions (tweets not relevant to the topic)

In both time periods, this category represented the majority of tweets: 74% of the sample before the shootings, and 37% of the sample after the shootings. These tweets were not related to the Atlanta spa shootings or topics influencing the narrative about attacks on Asians. Most of the tweets focused on appreciating Asian food, political opinions, and perspectives of popular culture and entertainment.

### Quantitative results

**Figure 1:**
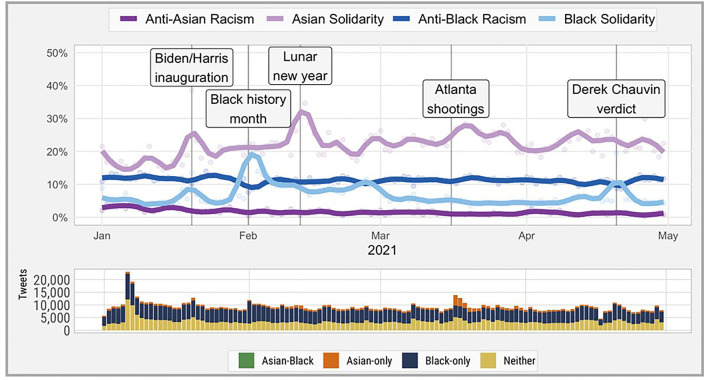
Impactful events, estimated rates of racism-related and solidarity messages on Twitter, and tweet volume January to April 2021, stratified by tweets containing Asian and Black keywords. Asian-Black denotes that tweets include both Asian and Black keywords. Solidarity speech was defined as hate speech scores <-3 and racist speech was defined as hate speech scores >0.5.

Overall, the quantitative data showed a temporary increase in solidary speech toward Asians after the Atlanta spa shooting. The top panel of Figure 1 shows the estimated rates of racism-related and solidarity tweets from January to April of 2021; the bottom panel shows the volume of tweets. The dark purple and dark blue lines show anti-Asian tweets and anti-Black tweets, respectively; these did not change noticeably across time. However, there were strong period effects for Asian solidarity and Black solidarity. The light purple line shows a 20% increase in solidarity-related tweets for Asians after the Atlanta spa shootings, from 22.7% 7 days before the shooting to 27.2% 3 days after (*p* < 0.001) (Figure 1). Within about 2 weeks, the increase in solidarity messaging returned to its earlier rate of ~20%.

For comparison, the study examined how hate and solidarity speech compared with another racial group with national race-related events. Specifically, we examined tweets with Black keywords; there were no detectable changes in solidarity-related sentiment following the Atlanta spa shootings. However, changes in Black solidarity speech were found with events related to the Biden/Harris presidential inauguration in January, the beginning of Black History month in February, and the announcement of Derek Chauvin's trial verdict on April 20. We identified no strong changes to rates of racism in tweets with Asian or Black keywords, but did find that tweets with Black keywords had higher rates of estimated racism compared to Asian keywords over the study period. Changes in tweet volume (lower subplot) were consistent with these findings, and further identified a spike in tweet volume during and immediately after the January 6 attack on the US capitol.

**Figure 2:**
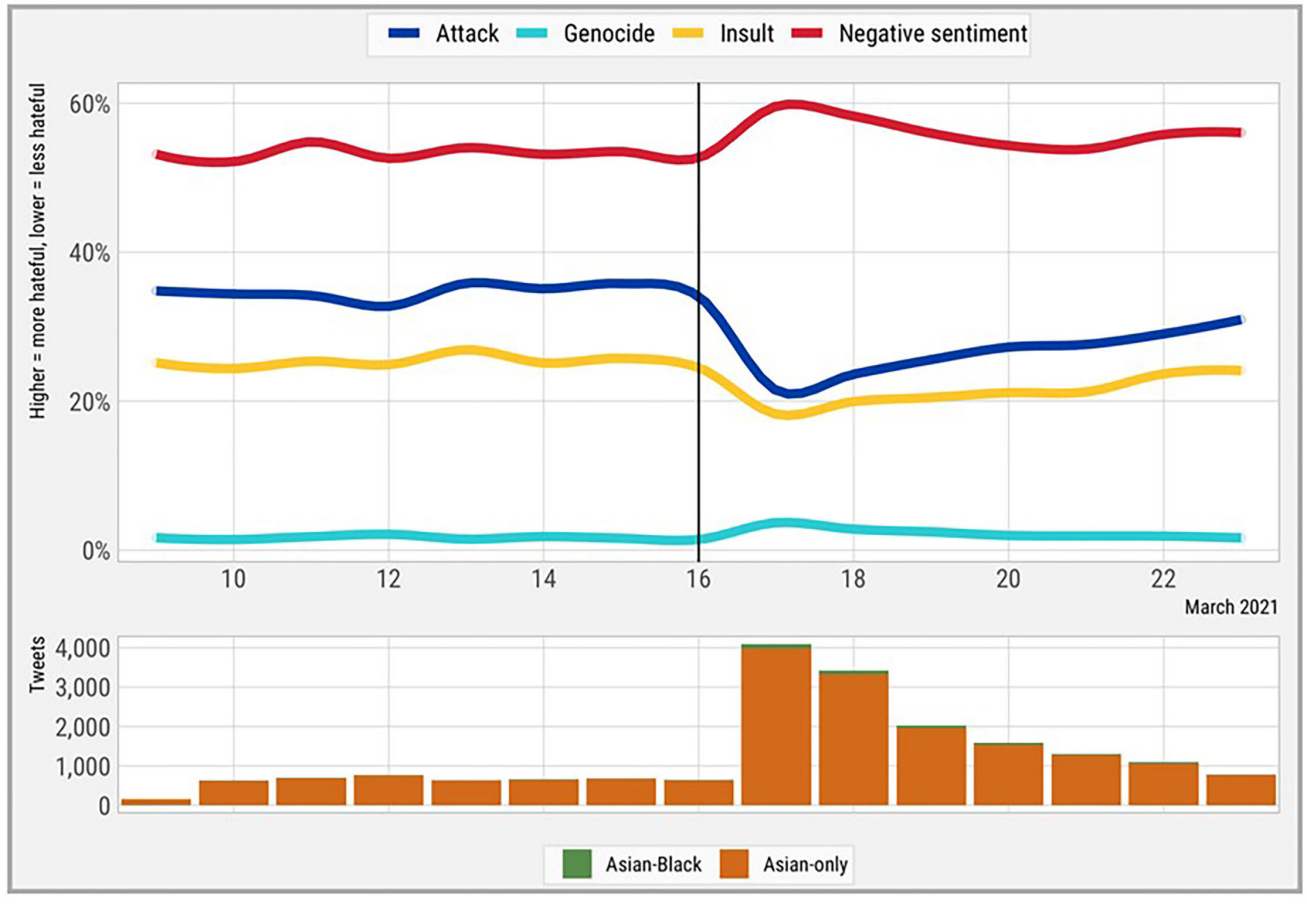
Longitudinal impacts of the Atlanta spa shooting on personal attacks, genocidal language, insults, and negative sentiment in Asian-keyword tweets.

In addition to the overall score, we examined trends in the individual components of hate speech (Table 1), with a focus on tweets containing Asian keywords (Figure 2). Around the Atlanta spa shooting, we identified a decrease in language that attacked Asians, a reduction in insults, an increase in negative sentiment tweets, and a very large but temporary increase in tweet volume lasting about 1 week.

We also evaluated hate speech score across selected themes identified from the qualitative content analysis for the period after the Atlanta spa shootings (Table 3). Tweets within the “solidarity” theme had the lowest average hate score, indicating that the model predicted those tweets to be in the supportive speech range of hate score (scores lower than −3). Other themes spanned the neutral range of the hate score (−1.5 to −0.5) and the positive speech or counter speech range (−3 to −1.5). The “Role of Racist Language” theme had the lowest average sentiment, indicating more negative sentiment, as well as more hateful average ratings on attack-defend, respect, and insult components.

**Table 3:** Comparison of the average hate speech score and average predicted ratings of key components after the Atlanta spa shootings (item rating range in parentheses) across themes, with chi-squared test of item rating variation across themes.

**Theme**	**Average hate score** **(SD)**	**Hate score** **(IQR)**	**Sentiment** **(SD)**	**Attack-** **defend** **(SD)**	**Respect** **(SD)**	**Insult** **(SD)**
Solidarity	−3.73 (1.03)	−4.5, −3.1	0.90 (1.10)	0.17 (0.43)	0.56 (0.86)	0.06 (0.30)
Condemn shootings	−1.86 (1.01)	−2.4, −1.2	2.74 (0.94)	0.67 (0.78)	2.00 (0.87)	0.53 (0.80)
Racism	−1.88 (0.90)	−2.4, −1.3	2.70 (0.77)	0.83 (0.76)	2.17 (0.84)	0.67 (0.86)
Language	−1.56 (0.97)	−2.1, −1	2.94 (0.75)	0.78 (0.89)	2.18 (0.93)	0.68 (0.89)
Intersectional	−1.90 (0.99)	−2.5, −1.3	2.60 (0.85)	0.62 (0.74)	2.01 (0.90)	0.55 (0.84)
Asian-Black	−1.95 (1.10)	−2.7, −1.1	2.56 (0.99)	0.81 (0.81)	2.01 (1.00)	0.73 (0.91)
Unrelated	−2.38 (1.15)	−3.1, −1.6	1.85 (1.06)	1.03 (0.44)	1.80 (1.05)	0.66 (0.80)
P-value			<0.0001	<0.0001	<0.0001	<0.0001

The possible values for the components of hate speech are 0–4 for the sentiment and respect items, and 0–3 for the attack-defend and insult items. This variation is due to combining response options based on fit statistics from the item response theory analysis, as reported in Kennedy et al. (24). Lower values indicate less hateful communication on that aspect of speech, while higher values indicate more hateful communication. See Supplementary Table 1 for examples of tweets at each end of a component's possible ratings.

## Discussion

This study leveraged data from Twitter and employed a mixed-methods approach to interrogate public discourse about race, racism, and solidarity following the tragic Atlanta spa shootings. The use of both quantitative and qualitative data maximized the breadth and depth of our analysis beyond what could be achieved using each approach alone.

Themes that emerged during the qualitative content analysis largely mirrored those identified by the machine learning hate speech model. After the Atlanta spa shootings, there was a spike in negative sentiment tweets. The qualitative analysis revealed this increase was mostly driven by the theme of condemning the shootings, and not anti-Asian rhetoric. These tweets expressed anger and frustration and exuded an overall negative tone. The quantitative findings also revealed a rise in solidarity speech, and this was consistent with that qualitative theme which became more prominent after the Atlanta spa shootings. Our study highlights the importance of utilizing a mixed methods approach.

Prior to and after the Atlanta spa shootings, we observed discussions about the intersections of racism and sexism experienced among Asian and Asian American women. After the shootings, we observed concerns expressed for the safety of Asian and Asian American women, based on their racial/ethnic and gender identities. These themes can be understood within the context of intersectionality theory. Intersectionality describes how multiple social identities converge at the individual-level to reflect multiple interlocking systems of oppression at the societal-level (28). Central to this theory is a recognition that individuals may be placed at heightened risk of discrimination and violence based on their membership in multiple structurally marginalized groups (e.g., race *and* gender). Scholars have extended intersectionality theory to describe the unique forms of racialized sexism and sexualized racism experienced by women of Asian descent. This form of intersectional vulnerability stems from a long history of sexual racial stereotyping of Asian women as “exotic geishas” or “sexually submissive,” among other harmful tropes (29). These stereotypes may lead to verbal, physical, and sexual violence committed against Asian American women (29). Set against this backdrop, our findings suggest that the Atlanta spa shootings exacerbated feelings of fear and anxiety among Asian American women and their loved ones.

Age is another identity that shapes people's lived experiences based on its intersections with race and gender, reflecting broader societal intersections of ageism, racism, and sexism (30). Indeed, several of the victims of the shooting were in their 60s and 70s, hate crimes against Asian American elders have received national attention (31), and many of the tweets in our analysis expressed concern for the safety of older Asian American women. This finding underscores the need to take a broad intersectional approach to better understand vulnerabilities individuals face based upon membership in multiple marginalized social categories.

The recent anti-Asian racism and attacks should not be seen as random or even unique to this pandemic, but rather as part of a long history of racialization and violence against Asians (4). Much of the research on this topic emphasizes the role of political and educational institutions, news, and media in perpetuating anti-Asian racism and violence (32). Several tweets in our study addressed the downplaying of racism against Asian people, including being silenced or a denial of the existence of anti-Asian racism. The model minority myth plays a key role. Its portrayal of all Asians as a successful monolith provides reason to ignore Asian American protests about discrimination and racial inequalities, and often renders them invisible in discussions of race and racism (33).

Our data suggest the importance of national support for the Asian American community. Tweets condemning racism have increased under the hashtag #StopAAPIHate (34). Previous research shows that solidarity through hashtags and counterhate messaging on social media is associated with decreases in hate speech across social media platforms (22). The Atlanta spa shootings catalyzed an increase in national awareness of racist messaging and Asian hate crimes in America and sparked national use of counterhate messaging to support the Asian American community.

Even with this renewed support of the Asian American community, our study found that tweets discussed the friction between Asian and Black people before and after the Atlanta spa shootings. Before the incident, the rise in Asian hate crimes seemed to be the impetus for Twitter users engaging in discussions about anti-Blackness. After the incident, there were specific debates about the lack of support from Asians for BLM, continued discussion of Black perpetrators attacking Asians, and the role of white supremacy in this overall tension. Within the manufactured racial hierarchy in the model-minority myth, white supremacy works to pit Asian and Black people against each other in a competition for resources (35) and introduces questions of merit and favor by who “belongs” more in the US (36).

As a powerful counter to the friction narrative, there were also calls for solidarity between Asian and Black communities. Previous research has emphasized the importance of contact as a tool to build intergroup empathy and action (37). Discussions on Twitter may help facilitate contact regardless of distance. Research has found that the most effective multiracial collaborations share several key characteristics: an ability to set aside narrow race-based politics and focus on larger issues, strong relationships between individuals and organizations, and the mobilization of the resources and communities of ethnic-specific organizations (38). Our study demonstrates social media as a platform where people actively express anti-racist sentiments through calls for solidarity and condemnation of white supremacy.

## Conclusion

Our study has several limitations. Twitter data represent what people are willing to express online. This expression may differ from in-person interactions and discussions. Future work can examine the extent to which Twitter-expressed solidarity is associated with community collective action and policymaking. In our content analysis, our research focused on the Atlanta spa shootings specifically, and future research can examine the broader topic of condemning racial violence. Compared to the general adult population in the US, adult Twitter users are younger and more educated (39), so the results may not be generalizable to the US adult population as a whole. Discussions related to race and racism vary by socio-demographics characteristics. For example, greater education is associated with more egalitarian racial attitudes (40). In addition, discussions related to race and racism may vary by online platform. More extreme views may be expressed in other platforms (e.g., Reddit, 4Chan). Tweets are limited to 280 characters, precluding more nuanced discussions.

This study has several strengths. It uses temporal data and mixed methods to examine a racialized event, the 2021 Atlanta spa shootings. This is the first application of the newly developed hate speech model to examine a specific event. Due to its foundation in item response theory, the measurement technology could assess social media speech in a comprehensive manner, with built-in correction for annotator bias, and place messages on a continuous spectrum of severity ranging from extreme hate speech to supportive solidarity speech.

Future research could investigate how conversational trends on Twitter were reflected in change in policies and practices. Future research could also investigate whether machine learning models can predict race-related hate crimes based on rising trends of hate speech on Twitter. Our analysis highlights some of the complexities of discrimination including that based on intersectional identities, the unique and shared struggles experienced by Black and Asian Americans, and the importance of nuanced evaluation of online speech. By understanding the conversations emerging from social media, we may learn about possible ways to produce solidarity promoting messages and dampen hate messages.

## Data availability statement

Twitter data were collected using Twitter's Application Programming Interface (API). Twitter's API is free and open to the public. Further inquiries can be directed to the corresponding author/s.

## Author contributions

SC, TN, EM, GG, CK, and QN contributed to the conception and design of the study. SC, TN, CK, SN, ET, LN, IY, MK, NT, and AS contributed to the data analysis and interpretation. SC, TN, and CK drafted the manuscript. GG, QN, and MVK contributed to the input, review, analysis, and editing. All authors critically reviewed the manuscript.
