# Supplementary material for: Solidarity and strife after the Atlanta spa shootings: A mixed methods study characterizing Twitter discussions by qualitative analysis and machine learning
Source: Front Public Health. 2023 Feb 7;11:952069. doi: 10.3389/fpubh.2023.952069 (PMC9941551; doi:10.3389/fpubh.2023.952069)
Supplement: Supplementary file 1 [file Table_1.DOCX]

**Online Supplemental Materials**

**Hate Speech Measure**

Solidarity speech was defined as hate speech scores less than -3 and racist speech was defined as hate speech scores greater than 0.5. Neutral speech is centered at approximately -1.5 on the hate speech scale, which is why these thresholds are not equidistant to zero. Each tweet was further scored on the components of hate speech, which provided the model’s prediction of how a human reviewer would have rated the tweet when following the annotation guidelines. For example, the probability of each of the 5 possible sentiment ratings (strongly positive to strongly negative) was estimated by the model for every tweet. The expected sentiment rating for a given comment could then be calculated by multiplying the comment’s sentiment probability distribution by the rating values (where strongly positive = 0 and strongly negative = 4) and taking the sum (i.e. a weighted average). Other components of hate speech include attack-defend (comments attacking or defending the group), genocide (calls for the deliberate killing of a large group of people), respectfulness (comment is respectful towards the group), and insult (comment is insulting towards the group). Examples of tweets at each end of a component’s possible ratings are presented in supplemental eTable 1.

**eTable 1. Example comments predicted to have a very low or very high rating on each hate speech component**

| **component** | **type** | **specific rating** | **predicted_prob** | **tweet** |
| --- | --- | --- | --- | --- |
| sentiment | min | sentiment_0 | 98% | So proud of you love it! Black Girl Magic at its finest |
| sentiment | min | sentiment_0 | 98% | Rest In Peace Cicely Tyson. She was an amazing actress, a truly beautiful black woman. Her legacy will live on forever |
| sentiment | min | sentiment_0 | 98% | As a UCLA season ticket holder I am so proud of the program and the support and love that they give to each other, and for the stand that they have taken against racism, inequality and injustice. The team, coaches, and staff are true leaders in the fight. We stand with you. |
| sentiment | max | sentiment_4 | 99% | I feel like shooting this n*gger |
| sentiment | max | sentiment_4 | 99% | Kill n*ggers |
| sentiment | max | sentiment_4 | 99% | I am gonna.make sure they lynch that african n*gger |
| respect | min | respect_0 | 97% | Today my daughter-in-law presented her curriculum on Anti-Racism. Another #proudmom moment! She was truly in her element! |
| respect | min | respect_0 | 97% | HAPPY BIRTHDAY throwback to the first time I met you and you were raising money for undocumented students. You are such an amazing light and have pushed me to grow as a person in so many ways!!! Hope you have a wonderful day !!! |
| respect | min | respect_0 | 97% | I’m getting tears in my eyes seeing @KamalaHarris get sworn in. What an amazing historic event! Giving my little African American niece someone amazing to look up to. The first multi racial WOMAN Vice President! You go girl! |
| respect | max | respect_4 | 98% | Kill n*ggers |
| respect | max | respect_4 | 98% | We gonna show you how to be a Racist from burning mississippi okay? N*GGER style and H*NKY Wh*re'ism okay. That's what we gonna teach you. Yeah you white H*NKY f*ggit for N*GGERS I am gonna blast you as a F*ggit! Then be prepared to fight. |
| respect | max | respect_4 | 98% | I am gonna.make sure they lynch that african n*gger |
| insult | min | insult_0 | 99% | As a POC immigrant woman I feel amazing today. I am so happy for all the little girls today seeing we can be anything if we are given a chance. My heart is full and the future looks bright |
| insult | min | insult_0 | 99% | Right back at you b sweetie...I have loved your work over the years.. I am not Jewish I just support...I have been in a Synagogue back at 16 as part of my Methodist confirmation class. It was beautiful. |
| insult | min | insult_0 | 99% | I’m so inspired by @Beyonce making history tonight!!!! Black girls we can live all our dreams!!!! We are powerful!!! |
| insult | max | insult_3 | 90% | NYPD not murdering you n*ggers fast enough. |
| insult | max | insult_3 | 90% | We gonna show you how to be a Racist from burning mississippi okay? N*GGER style and H*NKY Wh*re'ism okay. That's what we gonna teach you. Yeah you white H*NKY f*ggit for N*GGERS I am gonna blast you as a F*ggit ! Then be prepared to fight. |
| insult | max | insult_3 | 92% | Like the low class poor ass Somali- I would dump the goddamn LITTLE N*GGER F*GGITs in the garbage at child birth. Dump the damn N*GGER child in the trash if I had one come out of any p*ssy hollering I am the daddy. Bye trash that f*cker. Better yet give'em to a H*NKY |
| humiliate | min | humiliate_0 | 100% | I just can’t get over the history being made today. Dr. Biden as the First Lady with a career in education. Kamala Harris as the first female and POC VP. My daughter is alive for this moment. She gets to grow up seeing how capable women are. #Inauguration2021 |
| humiliate | min | humiliate_0 | 100% | Good morning to Black Women across the world. Y’all are the best hands down and deserve the world. You’re stronger than what you been through and I love you |
| humiliate | min | humiliate_0 | 100% | Match: Help refugee children. Support @RESCUEorg. The International Rescue Committee helps people whose lives and livelihoods are shattered by conflict and disaster to survive, recover and regain control of their future. |
| humiliate | max | humiliate_2 | 80% | Hispanics=snakes We dont need snakes in our servers. They dont need wifi or communication in mexico. Send them to wars. Be careful. They are strong and breed quickly. Perfect opportunity for castration. Use animals if your scared. |
| humiliate | max | humiliate_2 | 81% | Teenage hump and rape the kid? Maid and sex slave for life?? |
| humiliate | max | humiliate_2 | 84% | Had the slave whore in a trashy motel using his a*s as I please loads of PNP . Make him beg for more with me next time use him up like the pnp c*m dump party wh*re I’m training him to be... pnp dope cloud whore in training . His a*s has no choice |
| status | min | status_0 | 100% | The panel was half Anglo-/half Francophone |
| status | min | status_0 | 100% | His a also an nigerian |
| status | min | status_0 | 100% | Steve Jobs was Syrian? |
| status | max | status_1 | 95% | The evidence is and has been there in plain sight. The question that begs an answer is why anyone with an IQ in excess of a root vegetable believes anything this tribe of urban hillbilly grifters says or does. #TrumpIsTrash |
| status | max | status_1 | 95% | More idiocy from our inbred hillbilly. |
| status | max | status_1 | 95% | Now we do this except to swamp people and other exotic forms of white trash |
| dehumanize | min | dehumanize_0 | 100% | A black woman, made history. Changed history. Is history. Stacey Abrams, thank you |
| dehumanize | min | dehumanize_0 | 100% | #HappyBirthdayShakira Shakira is pure talent. She can play instruments she can dance and she can sing. She has transcended borders and has become a hopeful icon of global multiculturalism. #shakira #musical #latina #dance #singer #colombiagerl #Colombia #singersongwriter |
| dehumanize | min | dehumanize_0 | 100% | On this historic morning I would like to thank everyone who fought this battle. Particularly black women. You are rock stars! Never forget how important every single vote is. Decency prevailed! If we work together we can do anything. |
| dehumanize | max | dehumanize_1 | 94% | They don't care if Biden is actually a racist. They are just like a troop of monkeys throwing poop at the walls. |
| dehumanize | max | dehumanize_1 | 94% | Let's dehumanize them, like they take such glee in doing to others(POC)... Buncha monkeys! Animals! Savages! Can't help themselves, it's in their genes! So athletic, but not very smart. Watch your women around these beasts! |
| dehumanize | max | dehumanize_1 | 95% | The Puerto Rican problem did speak facts about the gargantuan wooly wildebeest that critter is forever a miserable cow spewing dark trash out that corrupted mouth piece |
| violence_phys | min | violence_phys_0 | 100% | Shocked to hear of the passing of Dr Nemam Ghafouri, Founder of Joint Help for Kurdistan. She gave up everything in Europe to come to Iraq to help #Yazidi women and kids. This was us a few days before her birthday in 2019. A mother to so many at this refugee camp. |
| violence_phys | min | violence_phys_0 | 100% | I just can’t get over the history being made today. Dr. Biden as the First Lady with a career in education. Kamala Harris as the first female and POC VP. My daughter is alive for this moment. She gets to grow up seeing how capable women are. #Inauguration2021 |
| violence_phys | min | violence_phys_0 | 100% | Match: Help refugee children. Support @RESCUEorg. The International Rescue Committee helps people whose lives and livelihoods are shattered by conflict and disaster to survive, recover and regain control of their future. |
| violence_phys | max | violence_phys_1 | 99% | Decapitate the Chinese government. |
| violence_phys | max | violence_phys_1 | 99% | i feel like lynching a KKK member |
| violence_phys | max | violence_phys_1 | 99% | F*ck George Floyd He’s a b*tch and a slave I’m going to Lynch him |
| genocide | min | genocide_0 | 100% | The bravery of the Burmese people taking to the streets to protest the murderous military terror regime and demanding a return to democracy is inspiring. Freedom-loving people everywhere stand with them. |
| genocide | min | genocide_0 | 100% | The resilience @YusraMardini has shown in her life is remarkably heroic. She swam for her life and the safety of others while escaping the Syrian civil war, and then proceeded to make history and swim for all refugees the following summer at the Rio Olympics #WomensHistoryMonth |
| genocide | min | genocide_0 | 100% | Match: Help refugee children. Support @RESCUEorg. The International Rescue Committee helps people whose lives and livelihoods are shattered by conflict and disaster to survive, recover and regain control of their future. |
| genocide | max | genocide_1 | 98% | Obviously we need to kill more Muslims now |
| genocide | max | genocide_1 | 98% | Time to bomb Syria. |
| genocide | max | genocide_1 | 98% | They all should just be killed. Avoid them reproducing. I don’t see a point in them being arrested just to join the Aryan inbred brotherhood in prison (if convicted). Just bring out the firing squad and kill all of them. |
| attack_defend | min | attack_defend_0 | 97% | I’m so sorry. We are coming together as a community to stop violence against Asians. We stand with you and you are loved and wanted. This is your home |
| attack_defend | min | attack_defend_0 | 97% | Please Stop The Asian Hate And Asian Are People Too #EndViolence #AsianLivesMatter #AsiansAreHuman #EndRacism |
| attack_defend | min | attack_defend_0 | 97% | No human is illegal!! Our immigrant families deserve protections and dignity all around. NOW: Immigrant rights are human rights - Join me and various others for a special Moral Monday on Immigration. EVERYBODY has a right to live! |
| attack_defend | max | attack_defend_3 | 89% | White people need to be exterminated |
| attack_defend | max | attack_defend_3 | 90% | I am gonna.make sure they lynch that african n*gger |
| attack_defend | max | attack_defend_3 | 90% | Obviously we need to kill more Muslims now |
| hatespeech | min | hatespeech_0 | 100% | Avi Lipkin: Israeli Election Update |
| hatespeech | min | hatespeech_0 | 100% | A couple hundred people gathered here at the Asian Gardens downtown. |
| hatespeech | min | hatespeech_0 | 100% | Speaker of Syrian parliament says the country will hold its presidential election on May 26. |
| hatespeech | max | hatespeech_1 | 98% | I swear to God I will kill one of u F*gget a*s n*gga over Dillan |
| hatespeech | max | hatespeech_1 | 98% | K*ll n*ggers |
| hatespeech | max | hatespeech_1 | 99% | I am gonna.make sure they lynch that african n*gger |
