# Supplementary material for: Solidarity and strife after the Atlanta spa shootings: A mixed methods study characterizing Twitter discussions by qualitative analysis and machine learning
Source: Front Public Health. 2023 Feb 7;11:952069. doi: 10.3389/fpubh.2023.952069 (PMC9941551; doi:10.3389/fpubh.2023.952069)
Supplement: Supplementary file 2 [file Table_2.DOCX]

**Online Supplementary Materials**

**eTable 1.** Race terms used in Atlanta spa shootings data collection.

| **Items** | **Race** |
| --- | --- |
| abg's  almond shaped eyes  an abg  asian  asian indian  asians  aznbbygirl  bamboo coon  bangalees  bangladeshi  bengalis  bhutanese  buddhahead  burmese  cambodian  cambodians  chamorro  chiegro  chinaman  chinese  ching chong  ching-chong  chink  chinks  chonky  coconut nigger  coolie  cracker jap  cunt-eyed  dink  dog muncher  dog-muncher  east asian  filipino  filipinos  fingernail rancher  fob  fresh off  gook  gookaniese  gookemon  gooky  guamanian  gyppo  indonesian  jap  japanese  karen people  korean  koreans  laotian  ling ling  little hiroshima  malayali  malaysian  mongolian  mongolians  nepalese  noodle nigger  north korean  oriental  orientals  rice burner  rice nigger  rice rocket  rice-nigger  sideways cooter  sideways pussy  slanted eye  slant-eye  slopehead  south asian  spearchucker  squinty  taiwanese  thai  thais  thin eyed  thin-eyed  tibetan  vietnamese  zipperhead  dothead  hindu  hindus  #blacklivesmatter  #blacklivesmatter  #blktwiter  blacktwitter  black twitter  #tamirrice  african american  african americans  african't  africoon  afro caribbean  afro-caribbean  bahamian  bahamians  banjo lip  bantu  biscuit lip  bix nood  black boy  black boys  black female  black girl  black girls  black history  black lives  black male  black man  black men  black woman  black women  blacks  blktwitter  bootlip  bounty bar  buffie  bumper lip  burnt cracker  burrhead  burundi  bush-boogie  carribean people  chain dragger  congo lip  congolese  coon  coonass  coon-ass  coontang  darkey  darkie  darky  egyptian  egyptians  ethiopian  ethiopians  field nigger  freddie gray  freddiegray  gable  ghanaian  ghetto  golliwog  groid  haitian  haitians  hotep  jamaican  jamaicans  jigaboo  jigarooni  jigga  jiggabo  jigger  jim crow  jordanian  kenyan  knuckle-dragger  liberian  moroccan  moroccans  moulie  mozambican  mud people  n word  negro  negroes  negros  nigerian  nigerians  nigette  nigga  niggah  niggas  nigger  niggers  nigglet  nigglets  niggress  niglet  nig-ngo  n-word  pickaninny  porch monkey  rwandan people  slave  slavery  slaves  somali  somalian  south african  sudanese  Tamir + Rice  tanzanian  tar baby  tar-baby  zambian  zimbabwean | Asian  Asian  Asian  Asian  Asian  Asian  Asian  Asian  Asian  Asian  Asian  Asian  Asian  Asian  Asian  Asian  Asian  Asian  Asian  Asian  Asian  Asian  Asian  Asian  Asian  Asian  Asian  Asian  Asian  Asian  Asian  Asian  Asian  Asian  Asian  Asian  Asian  Asian  Asian  Asian  Asian  Asian  Asian  Asian  Asian  Asian  Asian  Asian  Asian  Asian  Asian  Asian  Asian  Asian  Asian  Asian  Asian  Asian  Asian  Asian  Asian  Asian  Asian  Asian  Asian  Asian  Asian  Asian  Asian  Asian  Asian  Asian  Asian  Asian  Asian  Asian  Asian  Asian  Asian  Asian  Asian  Asian  Asian  Asian  Asian  Black  Black  Black  Black  Black  Black  Black  Black  Black  Black  Black  Black  Black  Black  Black  Black  Black  Black  Black  Black  Black  Black  Black  Black  Black  Black  Black  Black  Black  Black  Black  Black  Black  Black  Black  Black  Black  Black  Black  Black  Black  Black  Black  Black  Black  Black  Black  Black  Black  Black  Black  Black  Black  Black  Black  Black  Black  Black  Black  Black  Black  Black  Black  Black  Black  Black  Black  Black  Black  Black  Black  Black  Black  Black  Black  Black  Black  Black  Black  Black  Black  Black  Black  Black  Black  Black  Black  Black  Black  Black  Black  Black  Black  Black  black  Black  black  Black  Black  Black  Black  Black  Black  Black  Black  Black  Black  Black  Black  Black  Black  Black  Black  Black  Black  Black  Black |
